# First Detection and Molecular Analysis of *Leishmania infantum* DNA in Sand Flies of Kosovo

**DOI:** 10.3390/pathogens12101190

**Published:** 2023-09-24

**Authors:** Betim Xhekaj, Ina Hoxha, Katharina Platzgummer, Edwin Kniha, Julia Walochnik, Kurtesh Sherifi, Agim Rexhepi, Behlul Behluli, Vit Dvořák, Hans-Peter Fuehrer, Adelheid G. Obwaller, Wolfgang Poeppl, Jovana Stefanovska, Aleksandar Cvetkovikj

**Affiliations:** 1 Faculty of Agriculture and Veterinary, University of Prishtina “Hasan Prishtina”, Bulevardi “Bill Clinton”, 10000 Pristina, Kosovo; betim.xhekaj@uni-pr.edu (B.X.); kurtesh.sherifi@uni-pr.edu (K.S.); agim.rexhepi@uni-pr.edu (A.R.); behlul.behluli@uni-pr.edu (B.B.); 2 Department of Parasitology and Parasitic Diseases, Faculty of Veterinary Medicine-Skopje, Ss. Cyril and Methodius University in Skopje, Lazar Pop-Trajkov 5-7, 1000 Skopje, North Macedonia; jstefanovska@fvm.ukim.edu.mk; 3 Center for Pathophysiology, Infectiology and Immunology, Institute of Specific Prophylaxis and Tropical Medicine, Medical University Vienna, Kinderspitalgasse 15, 1090 Vienna, Austria; ina.hoxha@meduniwien.ac.at (I.H.); katharina.platzgummer@meduniwien.ac.at (K.P.); edwin.kniha@meduniwien.ac.at (E.K.); julia.walochnik@meduniwien.ac.at (J.W.); 4 Department of Parasitology, Faculty of Science, Charles University Prague, Viničná 7, 128 43 Prague, Czech Republic; vidvorak@natur.cuni.cz; 5 Department of Pathobiology, Institute of Parasitology, University of Veterinary Medicine Vienna, Veterinärplatz 1, 1210 Vienna, Austria; hans-peter.fuehrer@vetmeduni.ac.at; 6 Division of Science, Research and Development, Federal Ministry of Defence, Roßauer Lände 1, 1090 Vienna, Austria; adelheid.obwaller@bmlv.gv.at; 7 Department of Dermatology and Tropical Medicine, Military Medical Cluster East, Austrian Armed Forces, Brünner Straße 238, 1210 Vienna, Austria; wolfgang.poeppl@bmlv.gv.at

**Keywords:** Phlebotominae, *Larroussius*, leishmaniasis, PCR, Balkan, Kosovo

## Abstract

Phlebotomine sand flies (Diptera: Psychodidae) are the principal vectors of phleboviruses and *Leishmania* spp., the causative agents of leishmaniases. The Mediterranean sand fly fauna is diverse, and leishmaniasis, mainly caused by *Leishmania infantum,* is endemic in the Balkan countries. Despite recent entomological surveys, only some districts of Kosovo have been sampled for sand flies, with no proof/confirmation of *L. infantum*. This study aimed to gain further insights into the species composition of natural sand fly populations in previously unsampled districts and areas in Kosovo without reports of leishmaniasis and to detect *Leishmania* DNA in sand flies. A sand fly survey was conducted in 2022 in all seven districts of Kosovo. Collected females were screened for *Leishmania* DNA by PCR. Positive samples were sequenced and subjected to maximum likelihood analysis with reference sequences for further molecular characterization. The trapping activities at 114 different localities resulted in 3272 caught specimens, comprising seven sand fly species of two genera, namely *Phlebotomus neglectus*, *Ph. perfiliewi*, *Ph. tobbi*, *Ph. papatasi*, *Ph. simici*, *Ph. balcanicus* and *Sergentomyia minuta*. *Leishmania infantum* DNA was detected in three individual sand flies of *Ph. neglectus* and *Ph. perfiliewi.* This study provides the most extensive sand fly survey in Kosovo and reports the first record of *L. infantum* DNA in sand flies, indicating autochthonous circulation of *L. infantum*.

## 1. Introduction

Leishmaniasis comprises various zoo-anthroponotic disease patterns caused by the protozoan parasites *Leishmania* spp. (Kinetoplastida: Trypanosomatidae) and transmitted by infected female phlebotomine sand flies (Diptera: Psychodidae: Phlebotominae). According to the World Health Organization (WHO), leishmaniasis is one of the most important neglected tropical diseases. It is endemic in more than 98 countries on five continents, affecting approximately 12 million people worldwide. The annual incidence of visceral leishmaniasis (VL) is more than 58,000, and of cutaneous leishmaniasis (CL) is more than 220,000 [1,2,3]. In Europe, leishmaniasis is endemic in all Mediterranean countries. The most spread and epidemiologically significant is the transmission cycle of *Leishmania infantum,* which may cause disease in both human and animal hosts [4]. Despite various mammalian reservoir hosts, dogs remain a pivotal source in many situations where the transmission cycle of *L. infantum* occurs [5].

Even though Kosovo is considered a leishmaniasis-endemic country, data are still scarce and human as well as canine cases are clearly underreported [6]. However, recent data clearly highlight potential endemic *L. infantum* transmission cycles. While Lazri et al. [7] reported an overall seroprevalence of 1.7% in dogs from three districts of Kosovo in 2008, Xhekaj et al. [8] tested 18.4% of dogs from southwestern Kosovo positive for anti-*Leishmania* antibodies in 2016. A recent survey in all seven districts of Kosovo showed an overall seroprevalence of 4.21% in dogs [9], and only a few human leishmaniasis cases with related travel history to Kosovo have been published to date [10].

Of around 1000 described sand fly species, only a fraction are proven or suspected vectors of *Leishmania* parasites [11]. Species of the genus *Phlebotomus,* which are confined to the Afro-Eurasian regionaries, are the most important vectors in this region. The vectorial role of species within the genus *Sergentomyia* remains unproven and speculative [1]. Balkan countries exhibit a diverse sand fly fauna; in Kosovo, 13 species of the two aforementioned genera have been documented. Among these, previous studies reported several competent vectors for *L. infantum*, with *Phlebotomus* (*Larroussius*) *perfiliewi* Parrot, 1930, and *Ph*. (*La*.) *neglectus* Tonnoir, 1921 being the most abundant species [12,13]. Nevertheless, not all seven districts of Kosovo have been surveyed to date.

Epidemiological investigations and entomological surveys have always been crucial to better understand the endemicity of leishmaniasis foci and to determine the relationship between the vector species and the reservoirs involved in *Leishmania* transmission cycles [14]. Determining the geographical distribution of *L. infantum* in vectors is a prerequisite for creating a proper strategy and effective preventive measures against the spread of this disease. Such successful disease prevention and management primarily depend on the data obtained through continuous and up-to-date research. In this context, we carried out a large-scale entomological survey to determine the distribution of sand flies and the occurrence of *L. infantum* in the vectors. The survey covered all districts of Kosovo and deployed a PCR-based screening of the collected sand flies to assess the circulation of *Leishmania* in environments with potential zoonotic transmission cycles.

## 2. Materials and Methods

### 2.1. Study Area

**Figure 1:**
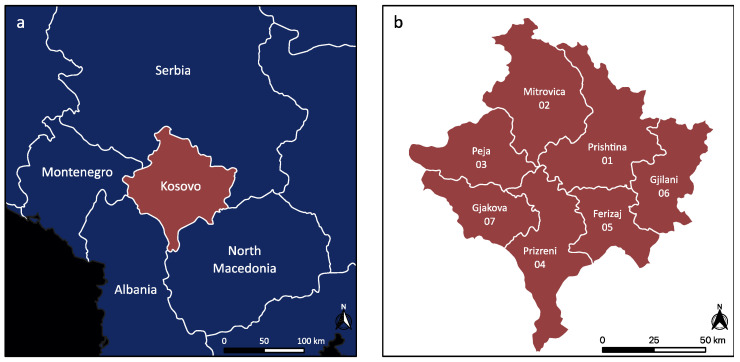
Map of Kosovo. The geographic position of Kosovo in the Balkan (**a**), seven districts of Kosovo (**b**). The Mediterranean Sea is shown in black on the left map.

The present study was conducted in the Republic of Kosovo, a landlocked country in the center of the Balkan Peninsula in South-Eastern Europe. It is located between latitudes 41° and 43° N and longitudes 20° and 22° E. The study area has a continental climate with Mediterranean and Alpine influences. Kosovo is divided into seven districts according to the law of Kosovo, namely Prishtina (01), Mitrovica (02), Peja (03), Prizreni (04), Ferizaj (05), Gjilani (06), and Gjakova (07) (Figure 1). In the countryside, agricultural activities include farming various animals (cattle, sheep and goats, poultry, and pigeons), and many dogs (stray, kept in private households, or as shepherd dogs) are widely present.

### 2.2. Entomological Sampling

An entomological survey was conducted between 1 July and 1 September 2022, in all seven districts of Kosovo. A total of 114 different locations were surveyed with up to three CDC miniature light traps (John W. Hock Company, Gainesville, FL, USA), resulting in 323 trap nights. The number of collection sites was as follows: 20 in Prishtina, 16 in Mitrovica, 16 in Peja, 15 in Prizreni, 15 in Ferizaj, 16 in Gjilani, and 16 in Gjakova district. Due to the restricted funding, sites sampled positive at first collection were not resampled, whereas some negative dog shelters were resampled. Sampling locations were chosen randomly, targeting dog shelters, cow farms, chicken farms, goat farms, sheep farms, and pigeon farms. Respective data on coordinates, farm type, and trap place were always registered (outdoor and indoor). Traps were operated overnight, set at sunset, and collected before sunrise the next day. Traps were placed one meter above the ground, outside and inside farms. After collection, the nets were placed immediately on dry ice, transported to the laboratory under cool conditions, and stored at −80 °C until dissection.

### 2.3. Morphological Identification of Sand Flies

Specimens were identified individually by morphology, with few exceptions. At locations with more than 100 individuals (five locations: 02/7, 03/7, 03/8, 04/9, and 07/8), we identified at least 8% of the catch individually, depending on the collection size. Therefore, the head and terminal segments of the abdomen of all caught sand fly specimens were dissected and slide-mounted in CMCP-10 mountant (Polysciences, Inc., Warrington, PA, USA). Identification was based on published morphological keys and descriptions of male genitalia, female spermatheca, and pharyngeal armature [15,16]. The remaining body parts were transferred to separate tubes for homogenization and nucleic acid extraction.

### 2.4. Molecular Analysis

For nucleic acid extraction, the specimens were homogenized in 500 µL (individual sand flies and pools with up to 15 specimens) or 1000 µL (pools with more than 15 specimens) Dulbecco’s Modified Eagle Medium (DMEM) supplemented with 20% bovine serum albumin, 1% penicillin/streptomycin, 10 µg/mL gentamicin, and 0.25 µg/mL amphotericin B (all from Gibco, Thermo Fischer Scientific). Two metal beads (3 mm diameter) were added to each 2.0 mL tube and homogenized with a TissueLyser bead mill (QIAGEN GmbH, Hilden, Germany) for 1 min of shaking at 30 Hz. The homogenate was cleared via centrifugation in a 4 °C benchtop centrifuge for 5 min at 14,000 rpm. All morphologically identified specimens were homogenized individually. The remaining unidentified specimens (at locations with more than 100 individuals) were sorted by sex, feeding status, and location and homogenized in pools of up to 30 specimens.

For DNA isolation, 200 µL supernatant was taken from an individual or pooled females, and DNA isolation was performed using the QIAmp^®^ DNeasy Blood and Tissue kit 250 (Qiagen, Hilden, Germany) by strictly following the manufacturer’s protocol with final elution in 100 µL. The remaining supernatants were stored at −80 °C for prospective RNA-based screenings.

### 2.5. Detection of Leishmania infantum DNA

Extracts of all individual and pooled females were screened for the presence of *Leishmania* spp. DNA. First, a sensitive nested-PCR protocol was applied, targeting the small subunit ribosomal ribonucleic acid (ssu) rRNA gene. For further species typing, two PCRs were applied, targeting the internal transcribed spacer 1 (ITS1) gene region and the cysteine protease B (cpb) gene region. In addition, all samples positive for *Leishmania* DNA were analyzed with a probe-based qPCR protocol targeting the kinetoplast DNA (kDNA) to obtain CT values. Primers and PCR protocols are given in Table 1.

All PCRs were performed with a 2× EmeraldAmp^®^ GT PCR Master Mix (Takara Bio Europe AB, Göteborg, Sweden) in a final volume of 25 µL with an Eppendorf Mastercycler (Eppendorf AG, Hamburg, Germany). Bands were analyzed using a Gel Doc™ XR + Imager (Bio-Rad Laboratories Inc., Hercules, CA, USA), cut out from the gel, and purified with an Illustra™ GFX™ PCR DNA and Gel Purification Kit (GE Healthcare, Buckinghamshire, UK). The samples were sent to Microsynth (Microsynth Austria GmbH, Vienna, Austria) for Sanger sequencing, and the obtained sequences from both strands were aligned with ClustalX 2.1 and edited with GeneDoc 2.7.0. The consensus sequences were blasted and uploaded in the NCBI sequence database (accession numbers: OR344780, OR345127–OR345132) and compared to reference sequences.

The qPCR was conducted using a TaqMan Universal PCR Master Mix (Applied Biosystems Inc., Waltham, MA, USA) in a final volume of 25 µL with a Bio-Rad CFX96 Touch Real-Time PCR Detection System Bio-Rad Laboratories, Inc., Hercules, CA, USA).

### 2.6. Molecular Identification of Leishmania-Positive Sand Flies

To confirm the morphological identification of sand fly specimens that tested positive for *Leishmania* DNA, a barcoding PCR targeting a 658-basepair (bp) fragment of the cytochrome c oxidase subunit I (COI) gene was performed using the primers LCO1490/HCO2198 following the protocol of Folmer et al. (1994) [22].

### 2.7. Molecular Characterization of Detected Leishmania DNA

Available *Leishmania* spp. reference sequences for comparison were downloaded from GenBank and aligned with the obtained sequences using ClustalX 2.1 for multiple alignment [23] and GeneDoc 2.7.0 [24] for manual editing and data analysis. For further clarification of species boundaries and to determine the discriminatory power of all three applied PCRs, pairwise distances and maximum likelihood (ML) analyses were calculated in MEGA X [25]. Based on the best-fit evolutionary model selection, the Kimura-2-parameter model with bootstrap support of 1000 replications was applied.

### 2.8. Mapping of Sand Fly Occurrence Data

Coordinates of sampling sites were georeferenced into a distribution map using Quantum GIS 3.4.11 [26]. Data on country, district, and municipality borders were taken from Natural Earth (naturalearthdata.com). First-level administrative divisions, Kosovo, 2015, were obtained from https://earthworks.stanford.edu/catalog/stanford-zh532mm5047 (accessed on 18 July 2023).

## 3. Results

### 3.1. Sand Fly Trapping

**Figure 2:**
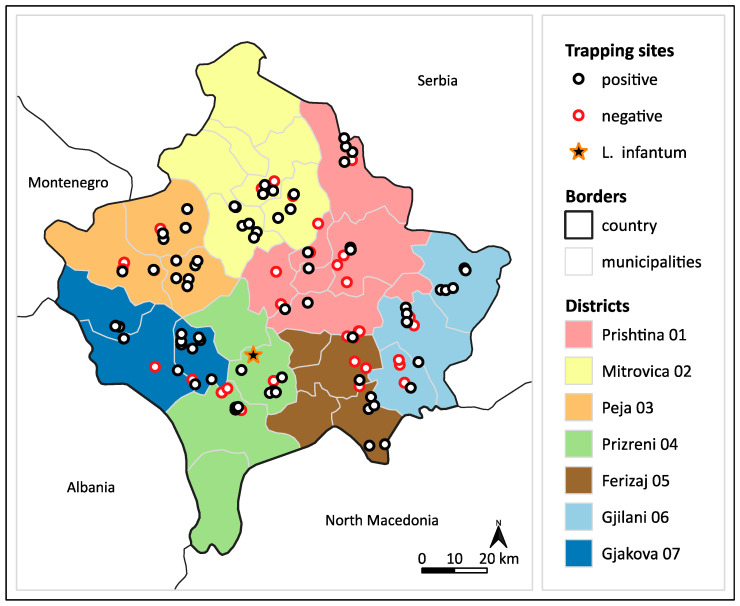
Map of positive and negative sand fly trapping sites in Kosovo. *L. infantum* positive site is indicated as a black star with an orange frame.

In total, 114 locations were sampled, of which 77 (67.5%) were positive for sand flies (Figure 2, Table S1). The number of sampling locations per region ranged from 15 to 20; the lowest capture rate was observed in Ferizaj 05 (7/15, 46.6%), whereas the highest was observed in Gjakova 07 (14/16, 87.5%).

Of 3272 trapped specimens, 2930 (89.5%) were female, and 342 (10.5%) were male. Of all females, 497 (497/2930; 17%) were engorged, and of all trapped specimens, 1149 were individually identified (from all locations with less than 100 trapped specimens), representing seven species in two genera, namely *Ph. perfiliewi* (715, 62.2%), *Ph. neglectus* (368, 32.0%), *Ph. tobbi* (27, 2.3%), *Ph. simici* (22, 1.9%), *Ph. balcanicus* (8, 0.7%), *Ph. papatasi* (2, 0.2%), and *Sergentomyia minuta* (7, 0.6%) (Table 2).

All other 2123 specimens originated from only five locations, namely 02/7 (45), 03/7 (146), 03/8 (925), 04/9 (715), and 07/8 (292). Of these, 372 specimens were individually identified as *Ph. perfiliewi* and *Ph. neglectus*, with *Ph. perfiliewi* always being the predominant species (Table 3).

The number of caught sand flies per district varied between 34 (Ferizaj 05) and 1299 (Peja 03). Gjakova had the most sand fly species (5 species), followed by Gjilani and Ferizaj (4 species), Peja and Prizreni (3 species), and Prishtina and Mitrovica (2 species) (Table 4).

*Phlebotomus perfiliewi* and *Ph. neglectus* were the only species found in all seven districts, whereas *Ph. balcanicus* was found only in Gjakova, and *Ph. papatasi* was found only in Peja (Table 4).

### 3.2. Leishmania DNA Screening

Of 2930 female specimens tested (915 individually and 2015 in 74 pools), three specimens (0.1%) were positive for *Leishmania* DNA: one *Ph. neglectus* and two *Ph. perfiliewi*. While the ssu rRNA (321 bp) and ITS1 (276 bp) PCRs gave positive results for all three samples, the cpb gene fragment (665 bp, 702 bp including primers) could only be amplified from the *Ph. neglectus* specimen. No nucleotide differences were observed between the respective ssu rRNA and cpb sequences of all three specimens. The kinetoplastid qPCR showed CT values of 24.9, 32.5, and 28.5 for the *Ph. neglectus* and the two *Ph. perfiliewi* specimens, respectively. Blast analysis results are given in Table 5. All three sand fly specimens positive for *Leishmania* DNA (3/828; 0.4%) originated from the same location (04/9): a cow farm in Semetisht village located in the Prizren district (Figure 2).

### 3.3. Molecular Characterization of L. infantum-Positive Samples

Reference sequences were obtained from GenBank for further characterization of *Leishmania* DNA sequences. In total, 17 (ssu rRNA), 17 (ITS1), and 15 (cpb) sequences were downloaded, aligned, and subjected to pairwise distance (pd) analysis. Gaps in the alignment were excluded from the analysis. The overall mean distances were 0.2% (S.E. = 0.2), 6.6% (S.E. = 0.9), and 8.0% (S.E. = 0.8) for ssu rRNA, ITS1, and cpb gene, respectively. Pairwise distances were lowest between *L. infantum* and *L. donovani* for all three gene fragments (0.06%/0.3%/0.8%) (Table 6). For ssu rRNA, low pd was observed, ranging from 0.06 to 0.5%. For ITS1 and cpb, pd was low between *L. infantum* and *L. donovani* (0.3% and 0.8%) but comparably high between other species (4.7 to 22.0% for ITS1, 8.5 to 13.4% for cpb) (Table 6).

**Figure 3:**
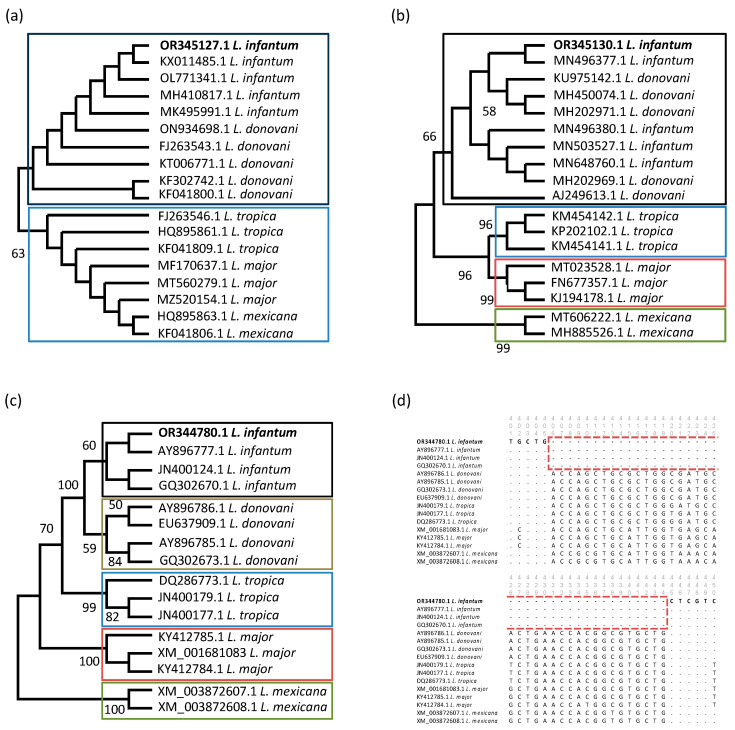
Discriminatory power of applied *Leishmania* PCRs. Maximum likelihood (ML) cladograms of *Leishmania* spp. based on three analyzed gene regions: ssu rRNA (**a**), ITS1 (**b**), and cpb (**c**). *L. mexicana* was used as an outgroup. Obtained sequences are shown, and they are marked with bold letters; colored squares indicate major clades observed. Bootstrap values over 50% are shown. Sequence alignment of *Leishmania* spp. showing the 39 bp deletion of *L. infantum* in the red dashed square (**d**).

All sequences used for pd calculations were included in the ML analysis, and *L. mexicana* was used as an outgroup for all three genes. The low discriminatory power of the ssu rRNA gene was shown as only two major clades were observed: clade I comprised *L. infantum* and *L. donovani*, whereas clade II comprised all other sequences of *L. tropica*, *L. major,* and *L. mexicana* (Figure 3a). ML analysis of ITS1 sequences revealed four clades, namely *L. donovani*/*infantum* (clade I), *L. tropica* (clade II), *L. major* (clade III), and *L. mexicana* (clade IV), thereby discriminating all species except *L. infantum* and *L. donovani* (Figure 3b). The highest discriminatory power was shown for the cpb gene, which divided the included sequences into five clades, including the discrimination of *L. infantum* and *L. donovani*, and supported by high bootstrap values (Figure 3c). The obtained cpb sequence in this study clustered with all other *L. infantum* sequences (clade I), and a 39 bp deletion was observed for all *L. infantum* sequences in the alignment between position 406 and 444, which is species-specific for *L. infantum* (Figure 3d). The complete alignment is given in Figure S1.

## 4. Discussion

This study represents the most extensive sand fly sampling effort in Kosovo, covering all seven districts and including the first detection of *L. infantum* DNA in sand fly vectors. *Leishmania* DNA was detected in two sand fly species: *Ph. neglectus* and *Ph. perfiliewi*.

Historical data on the distribution of sand flies in Kosovo are limited to only a few studies reporting the presence of ten species, namely *Ph. major*, *Ph. neglectus*, *Ph. perfiliewi*, *Ph. perniciosus*, *Ph. tobbi*, *Ph. sergenti*, *Ph. papatasi*, *Ph. simici*, *Ph. balcanicus*, and *S. dentata* [27,28]. Several recent studies confirmed the presence of some of these species and added three new records to the species list, namely *Ph. mascittii*, *Ph. alexandri,* and *S. minuta* [13,29,30]. In our study, we trapped seven different species of two genera, of which *Ph. perfiliewi* showed the highest trapping numbers and *Ph. neglectus* was the most widespread sand fly species found. This was also observed by Dvořák et al. [30], but not by Vaselek et al. [11], who reported *Ph. neglectus* to be the most trapped and most widespread species in their survey. While this is the most extensive sand fly survey in Kosovo, we found fewer sand fly species than in the other two studies. However, some species, such as *Ph. mascittii* and *Ph. alexandri*, which have not been detected in our study, were previously reported in very low numbers and at only a few locations. In our study, surveyed domestic and peridomestic sites as well as the sampling technique very much reflect the sampling sites of previous surveys that report the presence of these two species in Balkan countries [13,30]. We believe that our recorded absence of these species is based on their rareness in Kosovo and Balkan countries in general. Entomological sampling at or close to published locations of presence might further elucidate their actual distribution, particularly in southwestern parts of Kosovo.

Of all reported sand fly species in Kosovo, *Ph. neglectus*, *Ph. perfliewi*, *Ph. tobbi*, and *Ph. balcanicus* are proven vectors of *L. infantum* [4,31,32]. In our study, *Leishmania* DNA was detected in *Ph. neglectus* and *Ph. perfiliewi*, both important vectors of *L. infantum* in Mediterranean countries. Based on the available data, *L. infantum* is the predominant *Leishmania* species circulating in the Balkan region. However, not all studies discriminate between species within the *L. donovani* complex. We performed three different PCRs for species confirmation and specification and observed apparent differences in the specificity and sensitivity of the three applied PCRs. A sensitive nested PCR approach targeting the SSU rRNA gene (Table 1) was used for the initial screening of all samples, with a significantly lower discriminatory power [17,18]. A second PCR targeting the ITS1 region [19] confirmed all three positive samples; however, while discriminating all other *Leishmania* species as confirmed by ML analysis, the PCR lacks specificity to identify species within the *L. donovani* complex. A third species-specific PCR assay (cysteine protease B gene region) [20] for discriminating *L. infantum* and *L. donovani* gave a clear positive result only for the *Ph. neglectus* specimen, but only faint bands that could not be sequenced for the two *Ph. perfiliewi* specimens. We assumed that this is based on a lower sensitivity compared to the two other applied PCRs, and subsequent qPCR showed CT values of 24.9 for *Ph. neglectus* and higher values of 32.5 and 28.5 for the *Ph. perfiliewi* specimens. As stated by the authors [20], the cpb PCR can detect DNA yields of 50–100 pg. However, the sensitivity might be lower in mixed DNA samples. Therefore, establishing a nested PCR approach might be helpful for *L. donovani* complex typing from sand fly samples or other mixed samples with lower DNA yields compared to cultures or isolates.

All positive specimens originated from the same cow farm in Semetisht village (Prizren district), representing an overall prevalence of 0.1% of sand flies positive for *Leishmania* DNA. Vaselek et al. [11] detected *L. tropica* DNA in one *Ph. neglectus* specimen (118 tested) in Zhur (also Prizren district, approximately 35 km from Semetisht) close to the Albania border. Reports from neighboring countries such as Albania and Serbia have reported the presence of *L. infantum* DNA in sand flies. In Serbia, *L. donovani*/*infantum* DNA was detected in three engorged females of *Ph. papatasi,* which is not regarded as its vector and thus probably only accidentally picking up parasites that would not establish an infection in the sand fly. In total, 80 specimens were tested accounting for 4.0 % total positives [33]. In Albania, 15% of pools made of 422 *Ph. neglectus* specimens were positive for *Leishmania* DNA [34], and in another survey 3 of 55 mixed species pools including *Ph. neglectus* and/or *Ph. perfiliewi* were positive for DNA of the *L. donovani*/*infantum* complex [35]. However, these comparably higher prevalence rates compared to our study can be linked to specific sampling in areas with reported leishmaniasis cases. On the contrary, our survey represents a single-year, country-wide approach without targeted sampling. Interestingly, *Leishmania* spp. DNA has not yet been detected in sand flies from North Macedonia and Montenegro. However, leishmaniosis is circulating in dogs in all four neighboring countries of Kosovo [6].

The first report on canine leishmaniosis (CanL) in Kosovo caused by *L. infantum* was published in 2008, showing 1.7% seropositive dogs in three different municipalities, namely Prishtina, Ferizaj, and Gjakova [7]. The following study from 2016 reported the highest prevalence of seropositive dogs (21.6%) in the Prizren district, suggesting an ongoing transmission in this district [8]. The most recent seroprevalence survey on CanL in Kosovo was published in 2023, showing an overall seroprevalence rate in asymptomatic dogs of 4.21% by ELISA and 3.51% by IFAT in Kosovo [9].

While research on sand fly distribution and CanL in Kosovo has advanced recently, published data on human leishmaniasis are scarce. In recent years, the number of human leishmaniasis cases in Kosovo has been increasing (unpublished data). A previous study on the seroprevalence of anti-*Leishmania* antibodies in Austrian Armed Forces returning from Kosovo showed that 55 out of 261 soldiers (21.1%) had anti-*Leishmania* antibodies; of these, 13.8% showed positive, and 7.3% showed borderline results. The results demonstrate a considerable risk of exposure to *Leishmania* spp. in Kosovo, although some of the soldiers had been to other *Leishmania*-endemic countries before [36].

## 5. Conclusions

The detection of *L. infantum* DNA in *Ph. neglectus* and *Ph. perfiliewi* and previous seroprevalence data suggest that endemic leishmaniosis transmission cycles exist in Kosovo. In addition, the predominance of *Ph. neglectus* and *Ph. perfiliewi* and their universal occurrence in Kosovo corroborates their involvement in *L. infantum* transmission. To fully elucidate *Leishmania* transmission cycles in Kosovo, future studies should include domestic animals and wildlife in screening approaches and follow-up on autochthonous human cases with targeted sand fly surveillance in the respective regions.

## Figures and Tables

**Table 1:** PCR-based protocols for the detection of *Leishmania* DNA used in this study.

Target	Primer and Probe (5′-3′)	Fragment Size (bp)	Protocol	Reference
ssu rRNA (1st round)	R221 (GGTTCCTTTCCTGATTTACG)	~600 bp	94 °C/5 min; 15 cycles: 94 °C/30 s, 53 °C/30 s, 72 °C/30 s; 72 °C 10 min	[17]
	R332 (GGCCGGTAAAGGCCGAATAG)			
ssu rRNA (2nd round)	R223 (TCCCATCGCAACCTCGGTT)	~350 bp	94 °C 5 min; 32 cycles: 94 °C/30 s, 65 °C/30 s, 72 °C/30 s; 72 °C 10 min	[18]
	R333 (AAAGCGGGCGCGGTGCTG)			
ITS1	LITSR (CTGGATCATTTTCCGATG)	~320 bp	94 °C/5 min; 34 cycles: 94 °C/20 s, 53 °C/30 s, 72 °C/1 min; 72 °C/10 min	[19]
	5.8S (TGATACCACTTATCGCACTT)			
cpb	cpfF (CGTGACGCCGGTGAAGAAT)	~740– 780 bp	94 °C/5 min; 34 cycles: 94 °C/30 s, 62 °C/1min, 72 °C/1 min; 72 °C/10 min	[20]
	cpbR (CGTGCACTCGGCCGTCTT)			
kDNA ^a^	F (CTTTTCTGGTCCTCCGGGTAGG)	-	50 °C/10 min; 95 °C/10 min; 45 cycles: 95 °C/15 s, 60 °C/1 min	[21]
	R (CCACCCGGCCCTATTTTACACCAA)			
	Probe (FAM-TTTTCGCAGAAC GCCCCTACCCGC-TAMRA)			

^a^ qPCR

**Table 2:** Individually identified sand flies.

Species	Male	Female (Engorged)	Total	Percentage
*Ph. perfiliewi*	64	651 (110)	715	62.2%
*Ph. neglectus*	156	212 (61)	368	32.0%
*Ph. tobbi*	5	22 (2)	27	2.3%
*Ph. simici*	3	19 (4)	22	1.9%
*Ph. balcanicus*	4	4 (0)	8	0.7%
*Ph. papatasi*	1	1 (0)	2	0.2%
*S. minuta*	1	6 (2)	7	0.6%
Total	234	915 (179)	1149	100%

**Table 3:** Pooled sand flies at locations with more than 100 specimens.

Location	Total	Number	Species		Pooled
ID	Number	Identified	*Ph. perfiliewi*	*Ph. neglectus*	Specimens
02/7	133	88	74 (84.1%)	14 (15.9%)	45 (4 pools)
03/7	203	57	56 (98.3%)	1 (1.7%)	146 (9 pools)
03/8	1002	77	76 (98.7%)	1 (1.3%)	925 (32 pools)
04/9	828	113	87 (77.0%)	26 (23.0%)	715 (24 pools)
07/8	329	37	37 (100%)	0 (0.0%)	292 (11 pools)
total	2495	372	330 (88.7%)	42 (11.3%)	2123 (80 pools)

**Table 4:** Sand fly species per district.

Species	Prishtina	Mitrovica	Peja	Prizreni	Ferizaj	Gjilani	Gjakova	Total
*Ph. perfiliewi*	4	165	213	92	1	8	232	715
*Ph. neglectus*	54	61	12	107	24	53	57	368
*Ph. tobbi*	0	0	0	0	1	26	0	27
*Ph. simici*	0	0	0	0	8	13	1	22
*Ph. balcanicus*	0	0	0	0	0	0	8	8
*Ph. papatasi*	0	0	2	0	0	0	0	2
*S. minuta*	0	0	1	5	0	0	1	7
in mixed pools ^a^	0	45	1071	715	0	0	292	2123
total	58	271	1299	919	34	100	591	3272

^a^ only consisting of Ph. perfiliewi and Ph. neglectus.

**Table 5:** Blast analysis results of generated sequences.

	Blast Identity Compared to Reference Sequences			
Gene	*L. infantum*	*L. donovani*	*L. tropica*	*L. major*
ssu rRNA ^a^	100% (MN757921.1, MK495991.1)	100% (CP022642.1, ON934698.1)	99.69% (KF302745.1, GQ332363.1)	99.69% (MT560279.1) to 99.38% (MZ520154.1)
ITS1 ^a^	100% (MZ362379.1, MN503527.1)	100% (MH202970.1, OQ184729.1)	92.39% (KC679052.1) to 90.78% (KY963131.1)	91.80% (MT423523.1) to 86.51% (ON243845.1)
Cpb ^b^	100% (CP027841.1) to 97.89% (AY896778.1)	99.75% (EU637909.1) to 98.52% (AY896783.1)	90.86% (DQ286773.1) to 87.44% (JN400184.1)	90.15% (JN944175.1) to 88.92% (JN400175.1)

^a^ same *Leishmania* DNA sequences observed from all three sand fly specimens. ^b^ Blast algorithm neglects 39 bp deletion of *L. infantum*

**Table 6:** Mean pairwise Kimura-2-parameter genetic distances (%) between analyzed *Leishmania* species based on three genes: ssu rRNA, ITS1, and cpb.

*Leishmania* spp.	*L. infantum*	*L. donovani*	*L. tropica*	*L. major*	*L. mexicana*
*L. infantum*	-				
*L. donovani*	0.06/0.3/0.8	-			
*L. tropica*	0.3/4.7/9.3	0.4/5.0/9.9	-		
*L. major*	0.4/5.5/11.0	0.5/5.8/11.6	0.1/5.5/8.5	-	
*L. mexicana*	0.3/18.2/12.8	0.4/17.9/13.4	0.0/20.4/12.6	0.1/22.0/13.3	-

## Data Availability

All data are included in the article and Supplementary Material.

## Supplementary Materials

The following supporting information can be downloaded at: https://www.mdpi.com/article/10.3390/pathogens12101190/s1, Figure S1: Sequence alignment of *Leishmania* spp. based on a cpb gene fragment showing the 39 bp deletion of *L. infantum* in the red dashed square. Obtained sequences are marked with bold letters.; Table S1: Surveyed locations in Kosovo.
