# Supplementary material for: First Detection and Molecular Analysis of Leishmania infantum DNA in Sand Flies of Kosovo
Source: Pathogens. 2023 Sep 24;12(10):1190. doi: 10.3390/pathogens12101190 (PMC10610191; doi:10.3390/pathogens12101190)
Supplement: Supplementary file 1 [file pathogens-12-01190-s001.zip › Supplementary Figure S1_Alignment_cpb.pdf]

**OR344780.1 *L. infantum***	**A G G G C G C G T G C G G G T C G T G C T G G G C G T T C T C G G C G G T C G G C A A C A T C G A G**	**[50]**
AY896777.1 *L. infantum*	. . . .	[50]
JN400124.1 *L. infantum*	. . . .	[50]
GQ302670.1 *L. infantum*	. . . .	[50]
AY896786.1 *L. donovani*	. A	[50]
AY896785.1 *L. donovani*	. A	[50]
GQ302673.1 *L. donovani*	. A	[50]
EU637909.1 *L. donovani*	. A	[50]
JN400179.1 *L. tropica*	. . . . T . . . . G	[50]
JN400177.1 *L. tropica*	. . . . T . . . . G	[50]
DQ286773.1 *L. tropica*	. . . . T . . . . G	[50]
XM_001681083.1 *L. major*	. . . . T	[50]
KY412785.1 *L. major*	. . . . T	[50]
KY412784.1 *L. major*	. . . . T	[50]
XM_003872607.1 *L. mexicana*	. . . . T	[50]
XM_003872608.1 *L. mexicana*	. . . . T	[50]

**OR344780.1 *L. infantum***	**T C G C A G T G G G C C C G T G C C G G C C A C G G C T T G G T G A G C C T G T C G G A G C A G C A**	**[100]**
AY896777.1 *L. infantum*	. . . .	[100]
JN400124.1 *L. infantum*	. . . .	[100]
GQ302670.1 *L. infantum*	. . . .	[100]
AY896786.1 *L. donovani*	. . . .	[100]
AY896785.1 *L. donovani*	. . . .	[100]
GQ302673.1 *L. donovani*	. . . .	[100]
EU637909.1 *L. donovani*	. . . .	[100]
JN400179.1 *L. tropica*	. . . . T . . . . C . G C . . A C . G C	[100]
JN400177.1 *L. tropica*	. . . . T . . . . C . G C . . A C . G C . . . . A	[100]
DQ286773.1 *L. tropica*	. . . . T . . . . G C . . A C . G C	[100]
XM_001681083.1 *L. major*	. . . . G T . . . . A A G C . . . . G	[100]
KY412785.1 *L. major*	. . . . G T . . . . A A G C . . . . G	[100]
KY412784.1 *L. major*	. . . . G T . . . . A A G C . . . . G	[100]
XM_003872607.1 *L. mexicana*	G G . . . . T A . . T . . . . A G C	[100]
XM_003872608.1 *L. mexicana*	G G . . . . T A . . T . . . . A G C	[100]

**OR344780.1 *L. infantum***	**G**	**C**	**T**	**G**	**G**	**T**	**G**	**A**	**G**	**C**	**T**	**G**	**C**	**G**	**A**	**T**	**G**	**A**	**C**	**A**	**A**	**A**	**G**	**A**	**C**	**A**	**A**	**T**	**G**	**G**	**C**	**T**	**G**	**C**	**A**	**A**	**C**	**G**	**G**	**C**	**G**	**G**	**G**	**C**	**T**	**G**	**A**	**T**	**G**	**C**	**[150]**
AY896777.1 *L. infantum*	.	.	.	.	.	.	.	.	.	.	.	.	.	.	.	.	.	.	.	.	.	.	.	.	.	.	.	.	.	.	.	.	.	.	.	.	.	.	.	.	.	.	.	.	.	.	.	.	.	.	[150]
JN400124.1 *L. infantum*	.	.	.	.	.	.	.	.	.	.	.	.	.	.	.	.	.	.	.	.	.	.	.	.	.	.	.	.	.	.	.	.	.	.	.	.	.	.	.	.	.	.	.	.	.	.	.	.	.	.	[150]
GQ302670.1 *L. infantum*	.	.	.	.	.	.	.	.	.	.	.	.	.	.	.	.	.	.	.	.	.	.	.	.	.	.	.	.	.	.	.	.	.	.	.	.	.	.	.	.	.	.	.	.	.	.	.	.	.	.	[150]
AY896786.1 *L. donovani*	.	.	.	.	.	.	.	.	.	.	.	.	.	.	.	.	.	.	.	.	.	.	.	.	.	.	.	.	.	.	.	.	.	.	.	.	.	.	.	.	.	.	.	.	.	.	.	.	.	.	[150]
AY896785.1 *L. donovani*	.	.	.	.	.	.	.	.	.	.	.	.	.	.	.	.	.	.	.	.	.	.	.	.	.	.	.	.	.	.	.	.	.	.	.	.	.	.	.	.	.	.	.	.	.	.	.	.	.	.	[150]
GQ302673.1 *L. donovani*	.	.	.	.	.	.	.	.	.	.	.	.	.	.	.	.	.	.	.	.	.	.	.	.	.	.	.	.	.	.	.	.	.	.	.	.	.	.	.	.	.	.	.	.	.	.	.	.	.	.	[150]
EU637909.1 *L. donovani*	.	.	.	.	.	.	.	.	.	.	.	.	.	.	.	.	.	.	.	.	.	.	.	.	.	.	.	.	.	.	.	.	.	.	.	.	.	.	.	.	.	.	.	.	.	.	.	.	.	.	[150]
JN400179.1 *L. tropica*	.	.	.	.	.	.	.	.	.	.	.	.	.	.	.	.	.	.	.	.	.	.	.	.	.	.	.	.	.	.	T	.	.	.	C	G	.	.	.	.	.	.	.	.	.	.	.	.	.	.	[150]
JN400177.1 *L. tropica*	.	.	.	.	.	.	.	.	.	.	.	.	.	.	.	.	.	.	.	.	.	.	.	.	.	.	.	.	.	.	T	.	.	.	G	C	.	.	.	.	.	.	.	.	.	.	.	.	.	.	[150]
DQ286773.1 *L. tropica*	.	.	.	.	.	.	.	.	.	.	.	.	.	.	.	.	.	.	.	.	.	.	.	.	.	.	.	.	.	.	T	.	.	.	G	G	.	.	.	.	.	.	.	.	.	.	.	.	.	.	[150]
XM_001681083.1 *L. major*	.	.	.	.	.	.	.	.	.	.	.	.	.	.	.	.	C	.	.	G	T	G	.	.	.	.	.	.	.	.	T	.	.	.	G	G	.	.	.	.	.	.	.	.	.	.	.	.	.	.	[150]
KY412785.1 *L. major*	.	.	.	.	.	.	.	.	.	.	.	.	.	.	.	.	C	.	.	G	T	G	.	.	.	.	.	.	.	.	T	.	.	.	G	G	.	.	.	.	.	.	.	.	.	.	.	.	.	.	[150]
KY412784.1 *L. major*	.	.	.	.	.	.	.	.	.	.	.	.	.	.	.	.	C	.	.	G	T	G	.	.	.	.	.	.	.	.	T	.	.	.	G	G	.	.	.	.	.	.	.	.	.	.	.	.	.	T	[150]
XM_003872607.1 *L. mexicana*	.	.	.	.	.	.	.	.	.	.	.	.	.	.	.	.	.	.	.	.	T	G	A	.	.	G	.	.	.	.	.	.	.	.	.	G	.	.	.	.	.	.	.	.	.	.	.	.	.	.	[150]
XM_003872608.1 *L. mexicana*	.	.	.	.	.	.	.	.	.	.	.	.	.	.	.	.	.	.	.	.	T	G	A	.	.	G	.	.	.	.	.	.	.	.	G	.	.	.	.	.	.	.	.	.	.	.	.	.	.	.	[150]

**OR344780.1 *L. infantum***	**T**	**G**	**C**	**A**	**G**	**G**	**C**	**G**	**T**	**T**	**C**	**G**	**A**	**G**	**T**	**G**	**G**	**C**	**T**	**G**	**C**	**T**	**G**	**C**	**G**	**A**	**C**	**A**	**C**	**A**	**T**	**G**	**T**	**A**	**C**	**G**	**G**	**G**	**A**	**T**	**C**	**G**	**T**	**G**	**T**	**T**	**C**	**A**	**C**	**G**	**[200]**
AY896777.1 *L. infantum*	.	.	.	.	.	.	.	.	.	.	.	.	.	.	.	.	.	.	.	.	.	.	.	.	.	.	.	.	.	.	.	.	.	.	.	.	.	.	.	.	.	.	.	.	.	.	.	.	.	.	[200]
JN400124.1 *L. infantum*	.	.	.	.	.	.	.	.	.	.	.	.	.	.	.	.	.	.	.	.	.	.	.	.	.	.	.	.	.	.	.	.	.	.	.	.	.	.	.	.	.	.	.	.	.	.	.	.	.	.	[200]
GQ302670.1 *L. infantum*	.	.	.	.	.	.	.	.	.	.	.	.	.	.	.	.	.	.	.	.	.	.	.	.	.	.	.	.	.	.	.	.	.	.	.	.	.	.	.	.	.	.	.	.	.	.	.	.	.	.	[200]
AY896786.1 *L. donovani*	.	.	.	.	.	.	.	.	.	.	.	.	.	.	.	.	.	.	.	.	.	.	.	.	.	.	.	.	.	.	.	.	.	.	.	.	.	.	.	.	.	.	.	.	.	.	.	.	.	.	[200]
AY896785.1 *L. donovani*	.	.	.	.	.	.	.	.	.	.	.	.	.	.	.	.	.	.	.	.	.	.	.	.	.	.	.	.	.	.	.	.	.	.	.	.	.	.	T	.	.	.	.	.	.	.	.	.	.	.	[200]
GQ302673.1 *L. donovani*	.	.	.	.	.	.	.	.	.	.	.	.	.	.	.	.	.	.	.	.	.	.	.	.	.	.	.	.	.	.	.	.	.	.	.	.	.	.	.	.	.	.	.	.	.	.	.	.	.	.	[200]
EU637909.1 *L. donovani*	.	.	.	.	.	.	.	.	.	.	.	.	.	.	.	.	.	.	.	.	.	.	.	.	.	.	.	.	.	.	.	.	.	.	.	.	.	.	.	.	.	.	.	.	.	.	.	.	.	.	[200]
JN400179.1 *L. tropica*	.	.	.	.	.	.	.	.	.	.	.	.	.	.	.	.	.	.	.	.	.	.	.	.	.	.	A	.	.	.	.	.	A	.	.	.	.	.	.	C	.	A	.	.	.	.	.	.	.	.	[200]
JN400177.1 *L. tropica*	.	.	.	.	.	.	.	.	.	.	.	.	.	.	.	.	.	.	.	.	.	.	.	.	.	.	A	.	.	.	.	.	A	.	.	.	.	.	.	C	.	A	.	.	.	.	.	.	.	.	[200]
DQ286773.1 *L. tropica*	.	.	.	.	.	.	.	.	.	.	.	.	.	.	.	.	.	.	.	.	.	.	.	.	.	.	A	.	.	.	.	.	A	.	.	.	.	.	.	C	.	A	.	.	.	.	.	.	.	.	[200]
XM_001681083.1 *L. major*	.	.	.	.	.	.	.	A	.	.	.	.	.	.	.	.	.	G	.	.	.	.	.	.	.	.	A	.	.	.	.	.	A	.	.	.	.	.	.	C	.	.	.	.	.	.	.	.	.	.	[200]
KY412785.1 *L. major*	.	.	.	.	.	.	.	A	.	.	.	.	.	.	.	.	.	G	.	.	.	.	.	.	.	.	A	.	.	.	.	.	A	.	.	.	.	.	.	C	.	.	.	.	.	.	.	.	.	.	[200]
KY412784.1 *L. major*	.	.	.	.	.	.	.	A	.	.	.	.	.	.	.	.	.	G	.	.	.	.	.	.	.	.	A	.	.	.	.	.	A	.	.	.	.	.	.	C	.	.	.	.	.	.	.	.	.	.	[200]
XM_003872607.1 *L. mexicana*	.	.	.	.	.	.	.	.	.	.	.	.	.	C	.	.	.	.	.	.	.	.	.	.	A	.	A	.	.	.	C	.	A	.	.	.	.	.	C	A	.	C	.	.	.	A	.	.	.	.	[200]
XM_003872608.1 *L. mexicana*	.	.	.	.	.	.	.	.	.	.	.	.	.	C	.	.	.	.	.	.	.	.	.	.	A	.	A	.	.	.	C	.	A	.	.	.	.	.	C	A	.	C	.	.	C	A	.	.	.	.	[200]

**OR344780.1 *L. infantum***	**G A G A A G A G C T A C C C C - T A C A C G T C C G G C A A C G G T G A T G T G G C C G A G T G C T**	**[250]**
AY896777.1 *L. infantum*	. . . . -	[250]
JN400124.1 *L. infantum*	. . . . -	[250]
GQ302670.1 *L. infantum*	. . . . -	[250]
AY896786.1 *L. donovani*	. . C . . . . -	[250]
AY896785.1 *L. donovani*	. . . . -	[250]
GQ302673.1 *L. donovani*	. . . . -	[250]
EU637909.1 *L. donovani*	. . . . C	[250]
JN400179.1 *L. tropica*	. . . G . C . . . . - . . . G T . . . . A . . . C . . . . T . . . . . C	[250]
JN400177.1 *L. tropica*	. . A G . C . . . . - . . . G T . . . . A . . . C . . . . T . . . . . C	[250]
DQ286773.1 *L. tropica*	. . . G . C . . . . - . . . G T . . . . A . . . G . . . . T . . . . . C	[250]
XM_001681083.1 *L. major*	. . . . - . . . G T C . . . . C	[250]
KY412785.1 *L. major*	. . . . - . . . G T C . . . . C	[250]
KY412784.1 *L. major*	. . . . - . . . G T C . . . . C	[250]
XM_003872607.1 *L. mexicana*	. . . G . C . . . . - . . . G T . . . . C T . . . . . C	[250]
XM_003872608.1 *L. mexicana*	. . . G . C . . . . - . . . G T . . . . C T . . . . . C	[250]

**OR344780.1 *L. infantum***	**T G A A C A G C A G T A A A C T C G T T C C C G G C G C G C A A A T C G A C G G C T A C G T G A T G**	**[300]**
AY896777.1 *L. infantum*	. . . .	[300]
JN400124.1 *L. infantum*	. . . .	[300]
GQ302670.1 *L. infantum*	. . . .	[300]
AY896786.1 *L. donovani*	. . . .	[300]
AY896785.1 *L. donovani*	. . . .	[300]
GQ302673.1 *L. donovani*	. . . .	[300]
EU637909.1 *L. donovani*	. . . .	[300]
JN400179.1 *L. tropica*	C . . . . C . . . . T . . . . G . . . . A . . . C .	[300]
JN400177.1 *L. tropica*	C . . . . C . . . . T . . . . G . . . . T . . . C .	[300]
DQ286773.1 *L. tropica*	C . . . . C . . . . T . . . . G . . . . A . . . C .	[300]
XM_001681083.1 *L. major*	C . . . . G . . . . . . C . . . . . . T . . . . G . . . . G . . . . . . T C .	[300]
KY412785.1 *L. major*	C . . . . G . . . . . . C . . . . . . T . . . . G . . . . G . . . . . . T C .	[300]
KY412784.1 *L. major*	C . . . . G . . . . . . C . . . . . . T . . . . G . . . . G . . . . . . T C .	[300]
XM_003872607.1 *L. mexicana*	C . . . . G . G . . . . . . G T . . . T . . . . . G . . . . C . . . . . T . .	[300]
XM_003872608.1 *L. mexicana*	C . . . . G . G . . . . . . G T . . . T . . . . . G . . . . C . . . . . T . .	[300]

**OR344780.1 *L. infantum***	**A T C C C G A G C A A C G A A A C G G T T A T G G C T G C G T G G C T T G C G G A G A A T G G C C C**	**[350]**
AY896777.1 *L. infantum*	. . . .	[350]
JN400124.1 *L. infantum*	. . . .	[350]
GQ302670.1 *L. infantum*	. . . .	[350]
AY896786.1 *L. donovani*	. . . .	[350]
AY896785.1 *L. donovani*	. . . .	[350]
GQ302673.1 *L. donovani*	. . . .	[350]
EU637909.1 *L. donovani*	. . . .	[350]
JN400179.1 *L. tropica*	. . . G A A . . . . G . . . . A	[350]
JN400177.1 *L. tropica*	. . . G A A . . . . G . . . . A	[350]
DQ286773.1 *L. tropica*	. . . G A A . . . . G . . . . A	[350]
XM_001681083.1 *L. major*	. . G G A A . . . . G . . . . . G A . . . . A	[350]
KY412785.1 *L. major*	. . G G A A . . . . G . . . . . G A . . . . A	[350]
KY412784.1 *L. major*	. . G G A A . . . . G . . . . . G A . . . . A	[350]
XM_003872607.1 *L. mexicana*	. . . G G A . . . . G . . . . . A . . C G . . . . C . . . A	[350]
XM_003872608.1 *L. mexicana*	. . . G G A . . . . G . . . . . A . . C G . . . . C . . . A	[350]

**OR344780.1 *L. infantum***	**C A T C G C G A T T G C G G T C G A C G C C A G C T C C T T C A T G T C T T A C C A G A G C G G C G**	**[400]**
AY896777.1 *L. infantum*	. . . .	[400]
JN400124.1 *L. infantum*	. . . .	[400]
GQ302670.1 *L. infantum*	. . . .	[400]
AY896786.1 *L. donovani*	. . . .	[400]
AY896785.1 *L. donovani*	. . . . T	[400]
GQ302673.1 *L. donovani*	. . . . T	[400]
EU637909.1 *L. donovani*	. . . .	[400]
JN400179.1 *L. tropica*	. . . . T . . . . T . . . . C	[400]
JN400177.1 *L. tropica*	. . . . T . . . . G . . . . C	[400]
DQ286773.1 *L. tropica*	. . . . T . . . . C	[400]
XM_001681083.1 *L. major*	. . . . T . . . . T . . . . T	[400]
KY412785.1 *L. major*	. . . . T . . . . T . . . . T	[400]
KY412784.1 *L. major*	. . . . T . . . . T . . . . T	[400]
XM_003872607.1 *L. mexicana*	. . . . C . G . . . . C . . . A	[400]
XM_003872608.1 *L. mexicana*	. . . . C . G . . . . C . . . A	[400]

```
OR344780.1 L. infantum          T G C T G - - - - - - - - - - - - - - - - - - - - - - - - - - - - - - - - - - - - - - - C T C G T C [450]
AY896777.1 L. infantum          . . . . . - - - - - - - - - - - - - - - - - - - - - - - - - - - - - - - - - - - - - - - . . . . . . [450]
JN400124.1 L. infantum          . . . . . - - - - - - - - - - - - - - - - - - - - - - - - - - - - - - - - - - - - - - - . . . . . . [450]
GQ302670.1 L. infantum          . . . . . - - - - - - - - - - - - - - - - - - - - - - - - - - - - - - - - - - - - - - - . . . . . . [450]
AY896786.1 L. donovani          . . . . . A C C A G C T G C G C T G G C G A T G C A C T G A A C C A C G G C G T G C T G . . . . . . [450]
AY896785.1 L. donovani          . . . . . A C C A G C T G C G C T G G C G A T G C A C T G A A C C A C G G C G T G C T G . . . . . . [450]
GQ302673.1 L. donovani          . . . . . A C C A G C T G C G C T G G C G A T G C A C T G A A C C A C G G C G T G C T G . . . . . . [450]
EU637909.1 L. donovani          . . . . . A C C A G C T G C G C T G G C G A T G C A C T G A A C C A C G G C G T G C T G . . . . . . [450]
JN400179.1 L. tropica           . . . . . A C C A G C T G C G C T G G G A T G C C T C T G A A C C A C G G C G T G C T G . . . . . T [450]
JN400177.1 L. tropica           . . . . . A C C A G C T G C G C T G G T G A T G C T C T G A A C C A C G G C G T G C T G . . . . . T [450]
DQ286773.1 L. tropica           . . . . . A C C A G C T G C G C T G G G G A T G C T C T G A A C C A C G G C G T G C T G . . . . . T [450]
XM_001681083.1 L. major        . C . . . A C C A G C T G C A T T G G T G A G C A G C T G A A C C A C G G C G T G C T G . . . . . T [450]
KY412785.1 L. major            . C . . . A C C A G C T G C A T T G G T G A G C A G C T G A A C C A C G G C G T G C T G . . . . . T [450]
KY412784.1 L. major            . C . . . A C C A G C T G C A T T G G T G A G C A G C T G A A C C A T G G C G T G C T G . . . . . T [450]
XM_003872607.1 L. mexicana     . . . . . A C C G C G T G C A T T G G T A A A C A G C T G A A C C A C G G T G T G C T G . . . . . . [450]
XM_003872608.1 L. mexicana     . . . . . A C C G C G T G C A T T G G T A A A C A G C T G A A C C A C G G T G T G C T G . . . . . . [450]

OR344780.1 L. infantum          G G G T A C A A C A A G A C C G G T G G G G T T C C G T A C T G G G T G A T C A A G A A C T C G T G [500]
AY896777.1 L. infantum          . . . . . . . . . . . . . . . . . . . . . . . . . . . . . . . . . . . . . . . . . . . . . . . . . [500]
JN400124.1 L. infantum          . . . . . . . . . . . . . . . . . . . . . . . . . . . . . . . . . . . . . . . . . . . . . . . . . [500]
GQ302670.1 L. infantum          . . . . . . . . . . . . . . . . . . . . . . . . . . . . . . . . . . . . . . . . . . . . . . . . . [500]
AY896786.1 L. donovani          . . . . . . . . . . . . . T . . . . . . . . . . . . . . . . . . . . . . . . . . . . . . . . . . . . [500]
AY896785.1 L. donovani          . . . . . . . . . . . . . . . . . . . . . . . . . . . . . . . . . . . . . . . . . . . . . . . . . [500]
GQ302673.1 L. donovani          . . . . . . . . . . . . . . . . . . . A . . . . . . . . . . . . . . . . . . . . . . . . . . . . . . [500]
EU637909.1 L. donovani          . . . . . . . . . . . . . . . . . . . . . . . . . . . . . . . . . . . . . . . . . . . . . . . . . [500]
JN400179.1 L. tropica           T . . . . . . . . . G . . . T . . . . A . . . . . . . . . . . . . . . . . . . . . . . . . . . . . . [500]
JN400177.1 L. tropica           . . . . . . . . . . G . . . T . . . . A . . . . . . . . . . . . . . . . . . . . . . . . . . . . . . [500]
DQ286773.1 L. tropica           . . . . . . . . . . G . . . T . . . . A . . . . . . . . . . . . . . . . . . . . . . . . . . . . . . [500]
XM_001681083.1 L. major        . . . . . . . . . . T . . . T . . . . A . . . . . . . . . . . . . . . . . . . . . . . . . . . . . . [500]
KY412785.1 L. major            . . . . . . . . . . T . . . T . . . . A . . . . . . . . . . . . . . . . . . . . . . . . . . . . . . [500]
KY412784.1 L. major            . . . . . . . . . . T . . . T . . . . A . . . . . . . . . . . . . . . . . . . . . . . . . . . . . . [500]
XM_003872607.1 L. mexicana     . . . . . . G . . . T . . . T . . . . A . . . . . . . . . . . . . . . . . . . . . . . . . . . . . . [500]
XM_003872608.1 L. mexicana     . . . . . . G . . . T . . . T . . . . A . . . . . . . . . . . . . . . . . . . . . . . . . . . . . . [500]
```

**OR344780.1 *L. infantum***	**G G G T G A G G A C T G G G G C G A G A A G G G C T A C G T G C G C G T G G C C A T G G G G C T G A**	**[550]**
AY896777.1 *L. infantum*	. . . .	[550]
JN400124.1 *L. infantum*	. . . .	[550]
GQ302670.1 *L. infantum*	. . . .	[550]
AY896786.1 *L. donovani*	. . . .	[550]
AY896785.1 *L. donovani*	. . . . A G . .	[550]
GQ302673.1 *L. donovani*	. . . . A G . .	[550]
EU637909.1 *L. donovani*	. . . .	[550]
JN400179.1 *L. tropica*	. . . . A . . . . T . . . . A . . . . G . . .	[550]
JN400177.1 *L. tropica*	. . . . A . . . . T . . . . A . . . . G . . .	[550]
DQ286773.1 *L. tropica*	. . . . T . . . . A . . . . G . . .	[550]
XM_001681083.1 *L. major*	. . . . A . . . . G . . .	[550]
KY412785.1 *L. major*	. . . . A . . . . G . . .	[550]
KY412784.1 *L. major*	. . . . A . . . . G . . .	[550]
XM_003872607.1 *L. mexicana*	. . . . . G . . . . C . . . . T G . . . . . . G . . .	[550]
XM_003872608.1 *L. mexicana*	. . . . . G . . . . C . . . . T G . . . . . . G . . .	[550]

**OR344780.1 *L. infantum***	**A C G C G T G C C T G C T C A G T G A A T A C C C C G T G T C C G C G C A T G T G C C G C A G A G T**	**[600]**
AY896777.1 *L. infantum*	. . . .	[600]
JN400124.1 *L. infantum*	. . . .	[600]
GQ302670.1 *L. infantum*	. . . .	[600]
AY896786.1 *L. donovani*	. . . .	[600]
AY896785.1 *L. donovani*	. . . . G	[600]
GQ302673.1 *L. donovani*	. . . . T C C	[600]
EU637909.1 *L. donovani*	. . . . G	[600]
JN400179.1 *L. tropica*	. . . . C	[600]
JN400177.1 *L. tropica*	. . . . C	[600]
DQ286773.1 *L. tropica*	. . . . C	[600]
XM_001681083.1 *L. major*	. . . . C . . G G . . . . T . . . . T C	[600]
KY412785.1 *L. major*	. . . . C . . G G . . . . T . . . . . A . T C	[600]
KY412784.1 *L. major*	. . . . C . . G G . . . . T . . . . T C	[600]
XM_003872607.1 *L. mexicana*	. . . . G . G	[600]
XM_003872608.1 *L. mexicana*	. . . . G . G	[600]

**OR344780.1 *L. infantum***	**C**	**T**	**C**	**A**	**C**	**C**	**C**	**C**	**T**	**[609]**
AY896777.1 *L. infantum*	.	.	.	.	.	.	.	.	.	[609]
JN400124.1 *L. infantum*	.	.	.	.	.	.	.	.	.	[609]
GQ302670.1 *L. infantum*	.	.	.	.	.	.	.	.	.	[609]
AY896786.1 *L. donovani*	.	.	.	.	.	.	.	.	.	[609]
AY896785.1 *L. donovani*	.	.	.	.	.	.	.	.	.	[609]
GQ302673.1 *L. donovani*	.	.	.	.	.	.	.	.	.	[609]
EU637909.1 *L. donovani*	.	.	.	.	.	.	.	.	.	[609]
JN400179.1 *L. tropica*	.	.	T	C	.	.	.	.	.	[609]
JN400177.1 *L. tropica*	.	C	.	.	.	.	.	.	.	[609]
DQ286773.1 *L. tropica*	.	C	.	.	.	.	.	.	.	[609]
XM_001681083.1 *L. major*	.	C	.	.	.	.	.	.	.	[609]
KY412785.1 *L. major*	.	C	.	.	.	.	.	.	.	[609]
KY412784.1 *L. major*	.	C	.	.	.	.	.	.	.	[609]
XM_003872607.1 *L. mexicana*	G	C	.	G	.	.	.	.	.	[609]
XM_003872608.1 *L. mexicana*	G	C	.	G	.	.	.	.	.	[609]
